# Predictive factors influencing outcome of early cranioplasty after decompressive craniectomy: a outcome prediction model study

**DOI:** 10.3389/fneur.2024.1384320

**Published:** 2024-06-05

**Authors:** Zhongnan Yan, Xiaolei Li, Bin Xia, Chaolin Xue, Yuangang Wang, Hongmin Che, Dongqing Shen, Shiwen Guo

**Affiliations:** ^1^Department of Neurosurgery, Xi’an Gaoxin Hospital, Xi’an, Shaanxi, China; ^2^Department of Neurosurgery, The First Affiliated Hospital of Xi’an Jiaotong University, Xi’an, Shaanxi, China

**Keywords:** decompressive craniectomy, cranioplasty, outcome, prediction, model

## Abstract

**Objective:**

The timing of cranioplasty (CP) has become a widely debated topic in research, there is currently no unified standard. To this end, we established a outcome prediction model to explore the factors influencing the outcome of early CP. Our aim is to provide theoretical and practical basis for whether patients with skull defects after decompressive craniectomy (DC) are suitable for early CP.

**Methods:**

A total of 90 patients with early CP after DC from January 2020 to December 2021 were retrospectively collected as the training group, and another 52 patients with early CP after DC from January 2022 to March 2023 were collected as the validation group. The Nomogram was established to explore the predictive factors that affect the outcome of early CP by Least absolute shrinkage analysis and selection operator (LASSO) regression and Logistic regression analysis. Receiver operating characteristic (ROC) curve was used to evaluate the discrimination of the prediction model. Calibration curve was used to evaluate the accuracy of data fitting, and decision curve analysis (DCA) diagram was used to evaluate the benefit of using the model.

**Results:**

Age, preoperative GCS, preoperative NIHSS, defect area, and interval time from DC to CP were the predictors of the risk prediction model of early CP in patients with skull defects. The area under ROC curve (AUC) of the training group was 0.924 (95%CI: 0.867–0.980), and the AUC of the validation group was 0.918 (95%CI, 0.842–0.993). Hosmer-Lemeshow fit test showed that the mean absolute error was small, and the fit degree was good. The probability threshold of decision risk curve was wide and had practical value.

**Conclusion:**

The prediction model that considers the age, preoperative GCS, preoperative NIHSS, defect area, and interval time from DC has good predictive ability.

## 1 Introduction

DC is a surgical procedure that involves the removal of a portion of the skull and expansion of the dura mater. Its primary purpose is to rapidly and effectively decrease intracranial pressure, alleviate brainstem compression, and reduce the likelihood of life-threatening conditions (such as cerebral trauma, cerebral infarction, and cerebral hemorrhage) leading to mortality. This surgical intervention is widely regarded as the preferred treatment for refractory intracranial hypertensive disease (1).

Large-area skull defects resulting from DC can alter the intracranial environment, leading to brain tissue ischemia and hypoxia. These defects can also disrupt the physiological balance with blood, cause disorder in cerebrospinal fluid dynamics, and give rise to syndrome of the trephined. This syndrome is characterized by headache, dizziness, restlessness, and fear, and also includes focal neurological deficits, reduced or lost consciousness or even signs of herniation (2). CP aims to repair the structure and function of the missing skull, correct cerebrospinal fluid dynamic disorders, restore cerebrovascular reserve capacity and blood perfusion, support the soft tissue of the scalp, protect the brain, and improve the patient’s neurological symptoms, cognitive function, and appearance (3).

The timing of CP has become a hot topic in clinical trials in recent years, and the definition of “early” CP remains controversial. At the recent International Neurotraumatology Progress Conference, relevant experts reached a consensus and recommended that the timing of CP be defined as: ultra-early: < 6 weeks or 42 days after craniotomy and decompression; early: 6 weeks to 3 months after craniotomy and decompression; mid-term: 3–6 months after craniotomy and decompression; late: 6 months after craniotomy and decompression (4). In addition, scientists are mainly concerned about the complications and outcome (whether neurological function, cerebral blood flow, cerebral blood flow, etc. are improved) in the early stage (6 weeks to 3 months) or even very early stage (within 6 weeks) after CP. There are many research results, but there are also certain differences. An increasing number of studies believe that early CP (1–3 months) has no impact on complications such as postoperative infection and outcome (5). Some researchers have also demonstrated that early CP results in higher complication rates, affecting patient outcomes (6, 7).

Existing studies on early CP primarily focus on evaluating the surgical impact on clinical outcomes. However, there is a noticeable gap in utilizing pertinent clinical information prior to and during CP to predict the outcomes of patients with skull defects. This approach can enable timely intervention before early CP and establish a theoretical and practical foundation for determining the suitability of patients for early CP. Consequently, it can significantly mitigate the potential adverse consequences associated with early CP.

LASSO regression methods emphasize individual-level analysis. Applying a least squares penalty reduces some coefficients and sets others to zero, thus retaining the features most relevant to the dependent variable (8). This study aims to use LASSO regression analysis method to explore the predictive factors that affect the outcome of early CP, build a prediction model, and conduct model verification.

## 2 Materials and methods

### 2.1 Study population

**Figure 1:**
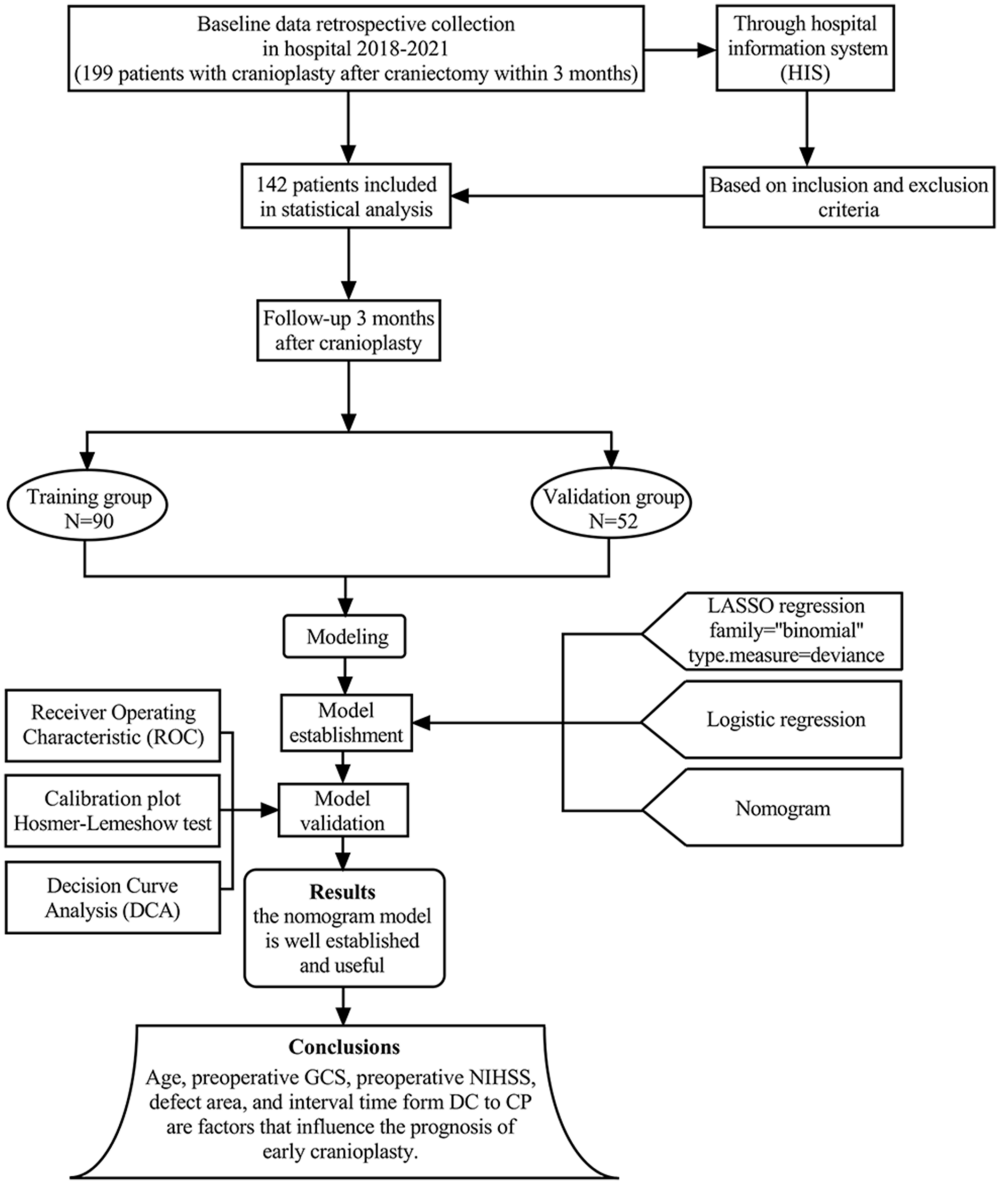
The flow chart shows the study process we have followed to build our predictive model, carry out the data collection, and conduct the model training and validation.

A total of 199 patients who were CP after DC within 3 months from January 2020 to March 2023 were screened. Among them, 142 patients who met the study criteria were enrolled, and their clinical data were reviewed through the Hospital Information System (HIS). The patients were divided into two groups. 90 patients from January 2020 to December 2021 as training group and 52 patients from January 2022 to March 2023 as validation group. A flow diagram is shown in Figure 1.

The inclusion criteria were as follows: (1) 18 years old ≤ age ≤ 80 years old; (2) Patients who underwent and unilateral DC due to cerebral hemorrhage, cerebral infarction, cerebral trauma, etc. upon admission; (3) CP should be performed within 1 to 3 months after DC (4). After DC, the intracranial pressure was normal, the scalp incision healed well, and there was no subcutaneous effusion, subdural effusion, and intracranial infection. No epilepsy occurred after DC for cerebral trauma (5). Before CP, head CT and MRI scans were performed to rule out intracranial hemorrhage and cerebral edema (6) First CP.

The exclusion criteria were as follows: (1) Age < 18 years old and > 80 years old. (2) Infection of intracranial and scalp incisions, intracranial hemorrhage, subcutaneous effusion, and subdural effusion before CP. (3) A poor functional outcome (mRs 5) before CP, in a coma or vegetative state and dead. (4) Patients with other serious complications or complications and dysfunction of important organs such as heart, liver, kidney, lung, etc. (5) Pregnant and breastfeeding women.

### 2.2 Collection of clinical information

All enrolled patients underwent DC due to cerebral hemorrhage, extensive cerebral infarction, or cerebral trauma. Within 3 months after DC, the neurosurgeon decides whether to perform CP based on the clinical situation and the opinions of the patient’s family. The following clinical data of the patients were collected: age, sex, BMI, past medical history (hypertension, diabetes, hyperlipidemia), etiology of DC (cerebral hemorrhage, cerebral infarction, cerebral trauma), preoperative GCS, preoperative NIHSS, side of DC, defect area, material, interval time form DC to CP, and operative time.

### 2.3 Outcome data

Neurosurgeons use the modified Rankin scale (mRs) to assess neurological recovery in patients 3 months after CP. The higher the value, the more severe the neurological deficit. The mRs score is as follows: 0 points: completely asymptomatic; 1 points: although there are symptoms, there is no obvious functional impairment, and all daily work and life can be carried out; 2 points: mild disability, unable to perform all pre-illness activities, but not required Assistance, able to take care of daily tasks; 3 points: moderate disability, needs some assistance but can walk independently; 4 points: moderate to severe disability, cannot walk independently, needs assistance. 5 points: Severe disability. The patient is bedridden, incontinent, completely dependent on others for daily living, and requires continuous care. Before early CP, patients were divided into two groups: one group with mRs 0–4 (good outcome group, 53 patients) and the other with mRs 5–6 (poor outcome group, 37 patients).

### 2.4 Statistical analyses

The data were analysed using statistical software SPSS 25.0 and R 4.3.2. The Kolmogorov–Smirnov test was performed to determine the normality of the measured data. Normally distributed data were expressed as (mean ± SD), and non-normally distributed data as median (interquartile range). Differences in normally distributed data were compared using the independent-samples t-test, and non-normally distributed data were compared using the Mann–Whitney U test. Categorical variables were compared using chi-square analysis. The ‘glmnet’ package and R software were applied to perform LASSO regression. The optimal influencing factors where the optimal penalty parameter λ value is located are determined through ten-fold cross-validation. On this basis, the statistically significant data in univariate logistic regression analysis were integrated into multivariable logistic regression to further construct a nomogram prediction model. The AUC was used to evaluate the model discriminability, the Hosmer-Lemeshow goodness of fit test was used to evaluate the model fit, and a calibration curve was drawn. DCA was performed to assess the clinical net benefit. If the *p* value is <0.1, the difference in means is considered statistically significant.

## 3 Results

### 3.1 Baseline characteristics

A total of 142 patients who underwent DC due to cerebral hemorrhage, cerebral infarction, or cerebral trauma and underwent CP within 3 months after DC were included in this study. Among the 90 cases in the training group, 53 cases (58.9%) had a good outcome and 37 cases (41.1%) had a poor outcome, see Table 1. There were 52 cases in the validation group. Comparing the basic information and factors of the two groups of patients between the training and validation groups, there was no statistically significant difference except for the defect area (*p* > 0.1). This shows that the two data sets are comparable, and the validation group data can be used to test the model effect, as shown in Table 2.

**Table 1:** Baseline characteristics of patients with early cranioplasty in training group.

Variables	Total (*n* = 90)	Good-outcome (*n* = 53)	Poor-outcome (*n* = 37)	Test value	*p*-value
Age (Mean ± SD)	51.09 ± 14.18	47.85 ± 12.25	55.73 ± 15.58	−2.682	0.009
Sex [*n* (%)]				2.350	0.125
Male	50 (55.6)	33 (62.3)	17 (45.9)		
Female	40 (44.4)	20 (37.7)	20 (54.1)		
BMI (Mean ± SD)	25.24 ± 3.39	25.62 ± 3.04	24.70 ± 3.82	1.271	0.207
Past medical history					
Hypertension [*n* (%)]				3.159	0.076
Yes	34 (37.8)	16 (30.2)	18 (48.6)		
No	56 (62.2)	37 (69.8)	19 (51.4)		
Diabetes [*n* (%)]				0.161	0.689
Yes	6 (6.7)	4 (7.5)	2 (5.4)		
No	84 (93.3)	49 (92.5)	35 (94.6)		
Hyperlipidemia [*n* (%)]				0.056	0.813
Yes	16 (17.8)	9 (17.0)	7 (18.9)		
No	74 (82.2)	44 (83.0)	30 (81.1)		
Etiology of DC					
Cerebral hemorrhage [*n* (%)]				2.777	0.096
Yes	30 (33.3)	14 (26.4)	16 (43.2)		
No	60 (66.7)	39 (73.6)	21 (56.8)		
Cerebral infarction [*n* (%)]				0.493	0.483
Yes	7 (7.8)	5 (9.4)	2 (5.4)		
No	83 (92.2)	48 (90.6)	35 (94.6)		
Cerebral trauma [*n* (%)]				1.474	0.225
Yes	53 (58.9)	34 (64.2)	19 (51.4)		
No	37 (41.1)	19 (35.8)	18 (48.6)		
Preoperative GCS [*n* (%)]				23.720	<0.001
<10	34 (37.8)	9 (17.0)	25 (67.6)		
≥10	56 (62.2)	44 (83.0)	12 (32.4)		
Preoperative NIHSS [*n* (%)]				20.710	<0.001
<20	50 (55.6)	40 (75.5)	10 (27.0)		
≥20	40 (44.4)	13 (24.5)	27 (73.0)		
Side of DC [*n* (%)]				0.242	0.623
Left	41 (45.6)	23 (43.4)	18 (48.6)		
Right	49 (54.4)	30 (56.6)	19 (51.4)		
Defect area [*n* (%)]				21.227	<0.001
<130 cm^2^	52 (57.8)	20 (37.7)	32 (86.5)		
≥130 cm^2^	38 (42.2)	33 (62.3)	5 (13.5)		
Materials [*n* (%)]				0.306	0.580
Ti	73 (81.1)	44 (83.0)	29 (78.4)		
PEEK	17 (18.9)	9 (17.0)	8 (21.6)		
Interval time form DC to CP [*n* (%)]				33.320	<0.001
<60 d	54 (60)	45 (84.9)	9 (27.0)		
≥60 d	36 (40)	8 (15.1)	28 (73.0)		
Oprerative time [*n* (%)]				0.301	0.583
<180 min	66 (73.3)	40 (75.5)	26 (70.3)		
≥180 min	24 (26.7)	13 (24.5)	11 (29.7)		

SD, Standard deviation; DC, Decompressive craniectomy; GCS, Glasgow coma scale; NIHSS, National institutes of health stroke scale; CP, Cranioplasty; Ti, Titanium; PEEK, Polyetheretherketone.

**Table 2:** The characteristics of training and validation groups.

Variables	Total (*n* = 142)	Training group (*n* = 90)	Validation group (*n* = 52)	Test value	*p*-value
Age (Mean ± SD)	50.81 ± 13.43	51.09 ± 14.18	50.52 ± 12.03	0.243	0.808
Sex [*n* (%)]				4.293	0.038
Male	88 (62.0)	50 (55.6)	38 (73.1)		
Female	54 (38.0)	40 (44.4)	14 (26.9)		
BMI (Mean ± SD)	50.81 ± 13.43	25.24 ± 3.39	25.54 ± 3.07	−0.525	0.601
Past medical history					
Hypertension [*n* (%)]				2.691	0.101
Yes	61 (43.0)	34 (37.8)	27 (51.9)		
No	81 (57.0)	56 (62.2)	25 (48.1)		
Diabetes [*n* (%)]				1.011	0.315
Yes	12 (8.5)	6 (6.7)	6 (11.5)		
No	130 (91.5)	84 (93.3)	46 (88.5)		
Hyperlipidemia [*n* (%)]				0.047	0.829
Yes	26 (18.3)	16 (17.8)	10 (19.2)		
No	116 (81.7)	74 (82.2)	42 (80.8)		
Etiology of DC					
Cerebral hemorrhage [*n* (%)]				0.150	0.699
Yes	49 (34.5)	30 (33.3)	19 (36.5)		
No	93 (65.5)	60 (66.7)	33 (63.5)		
Cerebral infarction [*n* (%)]				0.144	0.704
Yes	12 (8.5)	7 (7.8)	5 (9.6)		
No	130 (91.5)	83 (92.2)	47 (90.4)		
Cerebral trauma [*n* (%)]				0.342	0.559
Yes	81 (57.0)	53 (58.9)	28 (53.8)		
No	61 (43.0)	37 (41.1)	24 (46.2)		
Preoperative GCS [*n* (%)]				0.094	0.759
<10	55 (38.7)	34 (37.8)	21 (40.4)		
≥10	87 (61.3)	56 (62.2)	31 (59.6)		
Preoperative NIHSS [*n* (%)]				0.001	0.980
<20	79 (55.6)	50 (55.6)	29 (55.8)		
≥20	63 (44.4)	40 (44.4)	23 (44.2)		
Side of DC [*n* (%)]				0.358	0.549
Left	62 (43.7)	41 (45.6)	21 (40.4)		
Right	80 (56.3)	49 (54.4)	31 (59.6)		
Defect area [*n* (%)]				3.991	0.046
<130 cm^2^	73 (51.4)	52 (57.8)	21 (40.4)		
≥130 cm^2^	69 (48.6)	38 (42.2)	31 (59.6)		
Materials [*n* (%)]				2.608	0.106
Ti	109 (76.8)	73 (81.1)	36 (69.2)		
PEEK	33 (23.2)	17 (18.9)	16 (30.8)		
Interval time form DC to CP [*n* (%)]				0.033	0.857
<60 d	86 (60.6)	54 (60.0)	32 (61.5)		
≥60 d	56 (39.4)	36 (40.0)	20 (38.5)		
Oprerative time [*n* (%)]				0.048	0.827
<180 min	105 (73.9)	66 (73.3)	39 (75.0)		
≥180 min	37 (26.1)	24 (26.7)	13 (25.0)		

SD, Standard deviation; DC, Decompressive craniectomy; GCS, Glasgow coma scale; NIHSS, National institutes of health stroke scale; CP, Cranioplasty; Ti, Titanium; PEEK, Polyetheretherketone.

### 3.2 LASSO regression analysis

**Figure 2:**
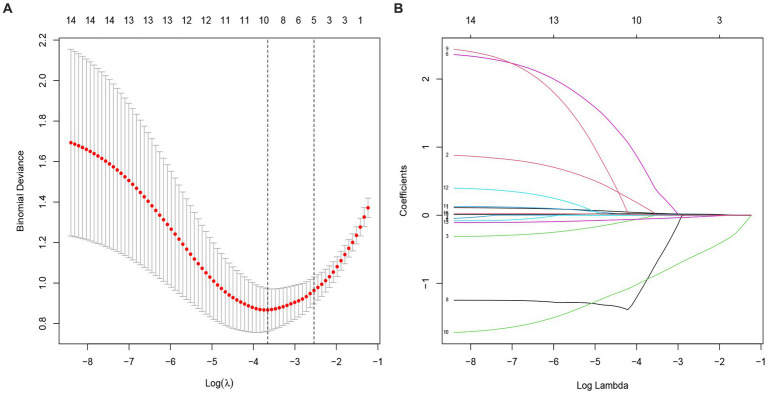
Selection of potential predictors using a LASSO regression model. **(A)** The optimal parameter (lambda) in the LASSO model was confirmed in the model by tenfold cross-validation of the minimum criterion. A dashed vertical line is drawn at the optimal value by using the smallest criterion (left dashed line) and one standard error of the smallest criterion (lambda.1SE) (right dashed line). **(B)** The model is optimal when λ increases to a standard error (lambda.1SE), and variables with nonzero coecients were screened out as potential predictors. It effectively decreased the 16 influencing factors to 5 as potential predictors.

All variables were included in the LASSO regression, and after the selection process, five variables were found to significantly influence the outcome of early CP. These variables include age, preoperative GCS, preoperative NIHSS, defect area, and interval time from DC to CP (Figures 2A,B).

### 3.3 Logistic regression analysis results of each factor that influences the outcome of patients with CP after DC within 3 months according mRs score

**Figure 3:**
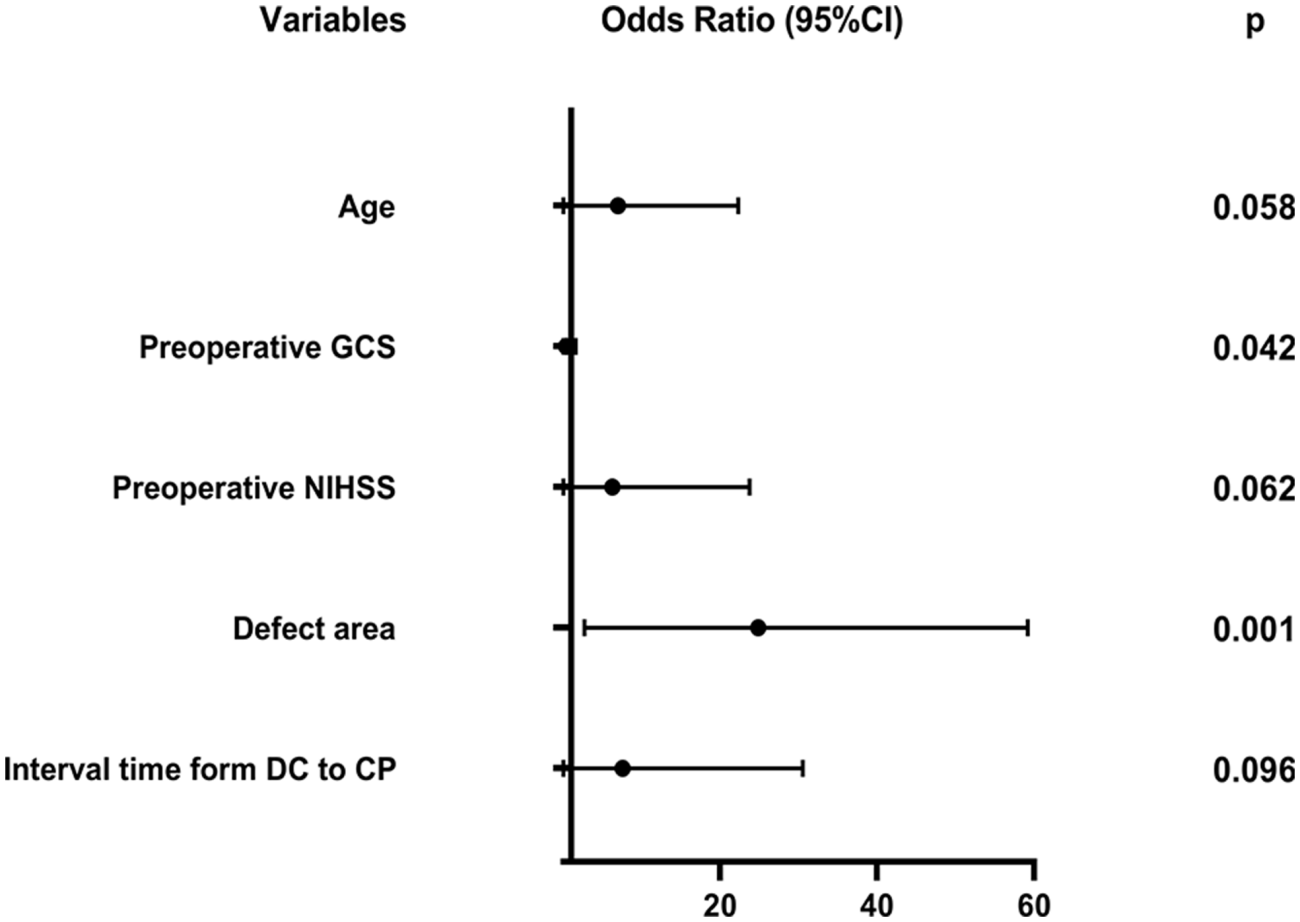
Multivariable logistic regression analysis.

Based on LASSO regression analysis, we conducted univariate and multivariable logistic regression analyses on the 5 potential predictors, resulting in the identification of 5 independent predictors. Table 3 and Figure 3 present the five independent predictors: age (OR: 4.105, 95% CI: 0.952–17.692, *p* = 0.058), preoperative GCS (OR: 0.180, 95% CI: 0.034–0.943, *p* = 0.042), preoperative NIHSS (OR: 3.654, 95% CI: 0.937–14.246, *p* = 0.062), defect area (OR: 12.678, 95% CI: 2.714–59.227, *p* = 0.001), and interval time from DC to CP (OR: 3.780, 95% CI: 0.789–18.106, *p* = 0.096).

**Table 3:** Univariate logistic regression analysis.

Variables	Univariate analysis			
	B	Wald	OR (95%CI)	*p*-value
Age	1.645	10.718	5.179 (1.935–13.861)	0.001
Preoperative GCS	−2.321	20.946	0.098 (0.036–0.265)	< 0.001
Preoperative NIHSS	2.117	18.758	8.308 (3.187–21.656)	< 0.001
Defect area	2.357	17.833	10.560 (3.536–31.534)	< 0.001
Interval time form DC to CP	2.862	19.427	17.500 (6.046–50.654)	< 0.001

GCS, Glasgow coma scale; NIHSS, National institutes of health stroke scale; DC, Decompressive craniectomy; CP, Cranioplasty.

### 3.4 Construction of the outcome risk prediction model in the training group

**Figure 4:**
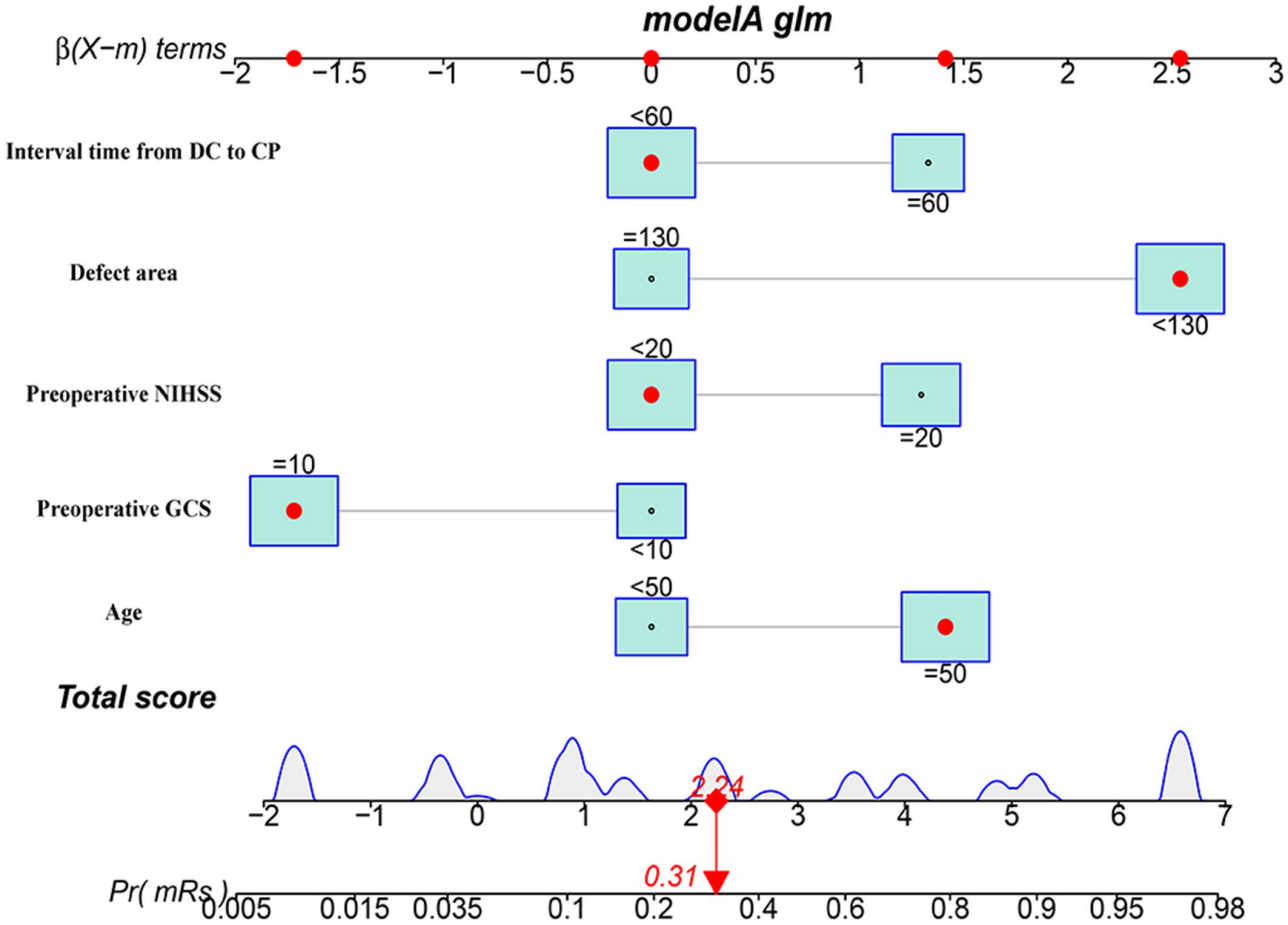
The nomogram model for predicting the risk of short-term prognosis in patients with early cranioplasty following DC.

We conducted a multivariate logistic regression analysis on the predictive variables selected through LASSO regression analysis. The analysis revealed that the factors included in the model were age, preoperative GCS, preoperative NIHSS, defect area, and interval time from DC to CP. To predict the probability of a poor outcome 3 months after early CP, we created a nomogram. The nomogram assigns a points value to each risk factor, and the sum of these scores corresponds to the probability of poor outcome in patients who underwent DCA higher total score indicates a greater likelihood of poor outcome (Figure 4).

### 3.5 ROC curve was constructed to evaluate model discrimination

**Figure 5:**
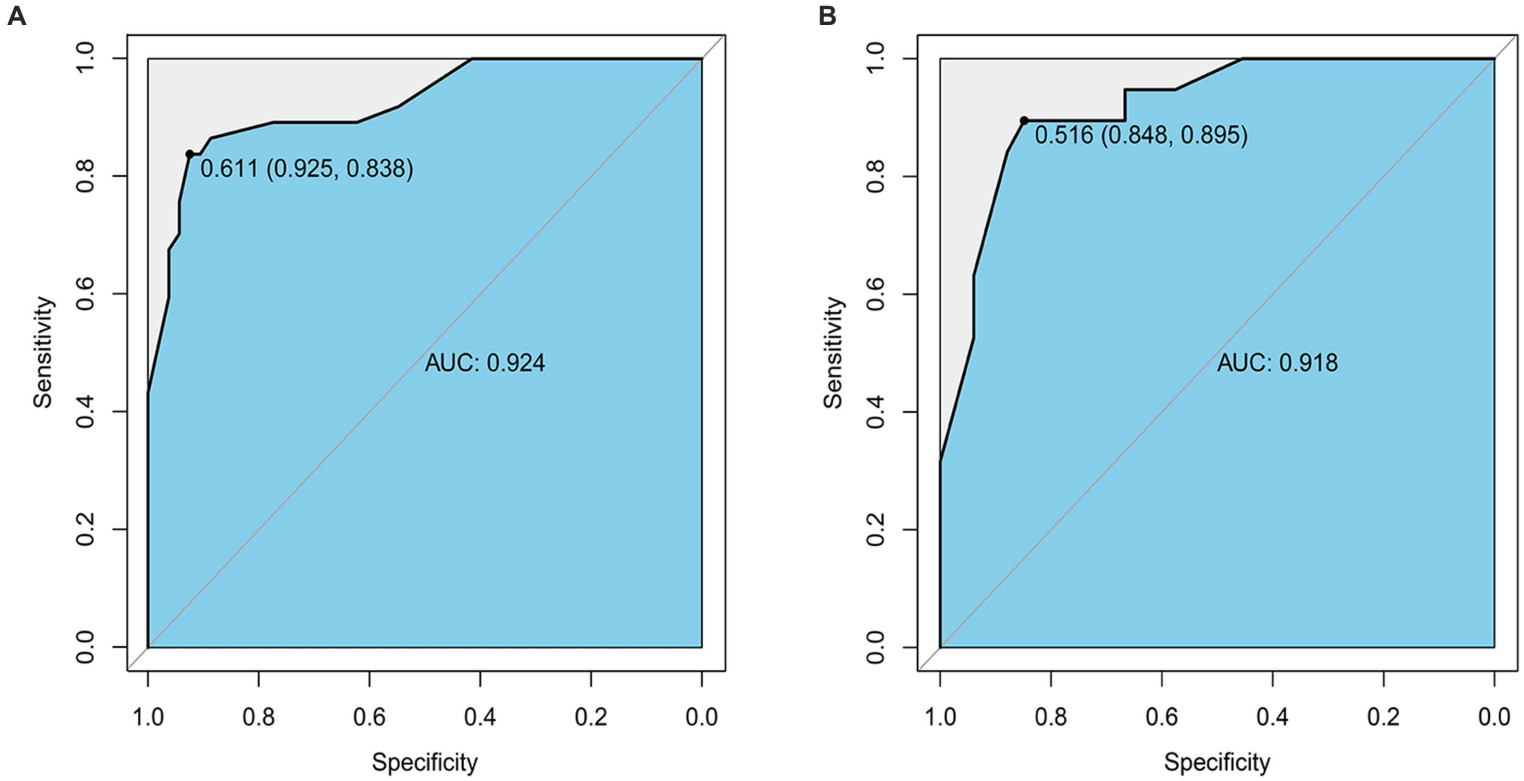
**(A)** The ROC curve of the Nomogram for the short-term prognosis of patients with early cranioplasty following DC in training group. **(B)** The ROC curve of the Nomogram for the short-term prognosis of patients with early cranioplasty following DC in validation group.

The nomogram prediction model’s accuracy was evaluated by drawing the ROC curve. In the training group, the area under the ROC curve was found to be 0.924 (95%CI: 0.867–0.980). This result was further validated using the validation group dataset, where the area under the ROC curve was 0.918 (95%CI: 0.842–0.993). These findings indicate that the prediction model demonstrates good discrimination in both the training and validation groups (Figures 5A,B).

### 3.6 Calibration curve was constructed to evaluate the prediction accuracy of the nomogram based on the prognostic model

**Figure 6:**
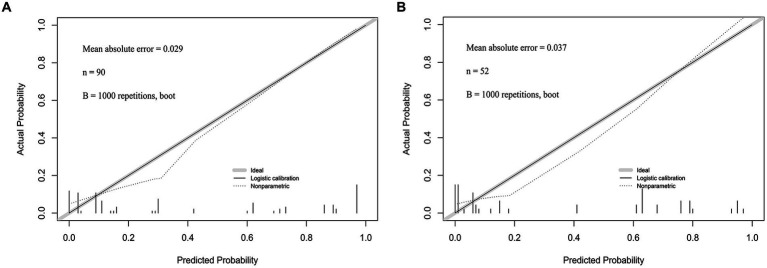
**(A)** The calibration curve of the Nomogram for the short-term prognosis of patients with early cranioplasty following DC in training group. **(B)** The calibration curve of the Nomogram for the short-term prognosis of patients with early cranioplasty following DC in validation group.

Calibration curves were plotted for the prediction models of patients with early CP after DC using both the training group and the validation group. The results demonstrated a close match between the actual and predicted incidence rates. The mean absolute error in the Hosmer-Lemeshow goodness-of-fit test was found to be 0.029, indicating a high level of accuracy. Furthermore, the validation group data set confirmed that the predicted probabilities aligned well with the actual probabilities. The Hosmer-Lemeshow goodness-of-fit test revealed an average absolute error of 0.037 (Figures 6A,B).

### 3.7 DCA was applied to evaluate the net benefit of model

**Figure 7:**
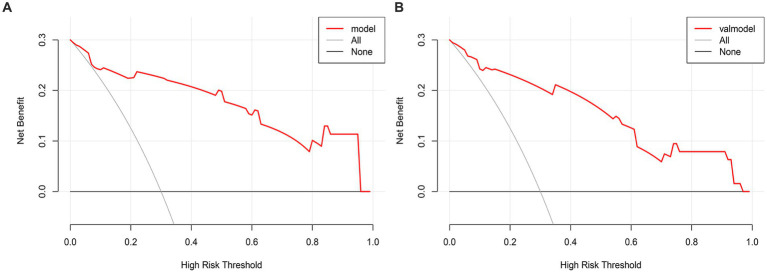
**(A)** The DCA curve of the Nomogram for the short-term prognosis of patients with early cranioplasty following DC in training group. **(B)** The DCA curve of the Nomogram for the short-term prognosis of patients with early cranioplasty following DC in validation group.

The DCA curve is plotted using the nomogram prediction model. The x-axis represents the threshold probability, while the y-axis represents the net income. The lines labeled ‘None’ and ‘All’ depict the two extreme scenarios. The results indicate that there are significant probability thresholds in both the training group and the validation group. This suggests that the nomogram prediction model holds clinical benefits and possesses valuable clinical application (Figures 7A,B).

## 4 Discussion

DC is a widely employed technique in neurosurgery to alleviate malignant intracranial hypertension resulting from brain tissue edema, brain injury, cerebral hemorrhage, extensive cerebral infarction, and other conditions. This approach effectively prevents life-threatening brain herniation, creates essential space and time for subsequent treatment, and contributes to enhancing the patient’s outcome (9).

After the primary craniocerebral disease is cured and the edema of the intracranial brain tissue subsides, the skull defect can lead to insufficient support for the brain tissue. This can affect the circulation of cerebrospinal fluid and the metabolism of brain tissue, resulting in secondary damage to the brain. Consequently, patients may experience various clinical and mental symptoms, including neurological dysfunctions like speech impairment, sensory loss in the body and limbs, reduced muscle strength, motor dysfunction, and epileptic seizures (10).

CP can restore the integrity of the skull, effectively increasing cerebral blood perfusion and improving cerebrospinal fluid circulation. It can also correct metabolic disorders of neuronal cells in the brain, stabilize intracranial pressure, and improve the patient’s clinical symptoms. Furthermore, CP can accelerate the recovery of brain function and effectively protect the patient’s brain tissue (11, 12).

In recent years, numerous studies have demonstrated the effectiveness of early CP (within 3 months after DC) in reducing long-term complications and improving treatment outcomes and prognosis for patients with traumatic brain injury (13). Our study focused on patients who underwent early CP and confirmed that a greater number of patients had a positive outcome at 3 months postoperatively compared to those with a poor outcome. Additionally, our study identified several significant factors that influence the 3-month prognosis after early CP, including age, preoperative GCS, preoperative NIHSS, defect area, and interval time from DC to CP. Based on these findings, we developed a prediction model to explore the relationship between potential clinical risk factors and the outcome of early CP in patients with skull defects. The ultimate goal of our research is to enhance the quality of life and survival outcomes for patients with skull defects following early CP.

Age is known to be correlated with the outcome of many diseases. As individuals age, their body’s compensatory capacity gradually decreases, leading to a decline in local blood supply. Consequently, the recovery period following an injury tends to be longer, and the incidence of complications tends to be higher due to a decrease in immunity (14). Additionally, a weakened immune system in older patients increases the likelihood of complications and higher incidence rates. Previous research, such as the NSQIP study, has recognized that advancing age independently contributes to adverse outcomes in patients undergoing CP (15). Furthermore, several studies have highlighted that individuals over the age of 50 face an elevated risk of epilepsy following CP (16). Our findings support the significant impact of age on the outcome of patients with skull defects who undergo early CP, with the patient’s outcome worsening as age increases. The reasons for these occurrences may be attributed to the following factors: (1) Elderly patients generally have lower cerebral blood flow and weaker brain pulse compared to young people (17). (2) Elderly individuals may experience varying degrees of neurodegeneration and brain atrophy. Combined with factors such as inadequate post-discharge care from family and social support, cognitive function decline in elderly patients can be accelerated (18). Therefore, it is crucial to conduct a comprehensive assessment of the patient’s physical condition and develop an individualized treatment plan prior to surgery.

Up to now, there are no unified regulations on the surgical indications for CP. Previous studies and relevant international consensus conferences have presented similar views, in cases of traumatic brain injury or stroke, bone removal is performed due to intracranial hypertension. For patients who undergo DC, it is essential that their neurological status and intracranial pressure are stable before CP, and there should be no systemic or intracranial infection (19). In this study’s training group, Eight patients exhibited severe disturbance of consciousness, as indicated by a GCS score of less than 9 points, accounting for 8.9% of the total patient population. Among these patients, more than 85% of the patients did not suffer from severe disturbance of consciousness, indicating that the selected patients met the basic criteria for CP. Furthermore, the optimal neurological recovery period for severe intracerebral injury and cerebral hemorrhage is 1 to 3 months after the injury, which aligns with the timeframe we chose for CP. This also explains why most patients did not experience severe disturbances of consciousness. The assessment of the admission GCS score is crucial in predicting the potential neurologic outcome of surgical procedures (20). In our study, we found that admission GCS score < 10 points is a significant factor affecting the outcome of early CP. Patients with GCS < 10 points often experience severe speech dysfunction, sensory impairment of the body and limbs, and muscle dysfunction. Additionally, these patients have a large area of intracranial damage and severe damage, leading clinicians to choose a relatively large bone flap during the operation. This can cause the brain tissue to shift and dent significantly with changes in gravity and atmospheric pressure when the patient sits or stands after surgery, resulting in a decrease in intracranial pressure. Conversely, when lying down, the effect of gravity is weakened, causing the brain tissue to bulge outside the cranial cavity. These changes in brain tissue position and shape can put the blood vessels in the cerebral cortex under constant stretching and twisting, leading to cerebral vasospasm and contraction, causing prolonged ischemia and hypoxia in the brain tissue’s corresponding arterial blood supply area, which hinders the recovery of neurological function (21).

The NIHSS score is a reliable indicator for assessing the short-term and long-term outcome of stroke (22). It is not typically used to assess the neurological status of patients with traumatic brain injury and CP following DC. However, if patients with traumatic brain injury exhibit symptoms of neurological deficits, such as alterations in consciousness, movement disorders, sensory impairments, etc., these symptoms may resemble those seen in stroke patients. In such cases, similar assessment tools can be utilized to evaluate their neurological status. In a previous study, the mean NIHSS at admission was compared between patients with ischemic stroke to determine the neurological outcomes (good outcome: mRs ≤ 3, poor outcome: mRs > 3) in patients with anterior circulation ischemic stroke. The results revealed that the mean NIHSS for posterior circulation stroke was significantly lower in the group with good functional outcomes, as compared to anterior circulation stroke (23). Wouter et al. (24) found that at 90 days after discharge, the main factor predicting patient outcome based on the initial severity of acute ischemic stroke and improvement within 24 h of admission was the NIHSS. The good outcome is defined as mRs 0–2. The results of LASSO and multivariate logistic regression analysis in this study showed that NIHSS ≥20 (OR = 3,654) is an independent factor that affects the short-term outcome of early CP. Patients with consciousness disorders are in a passive protection state. After CP, the impact on the skin flap from external forces cannot be relieved quickly, which may easily lead to secondary brain damage (25). Therefore, high NIHSS may be a reason for the poor outcome of patients with early CP.

Few previous studies have examined the effects of skull defect size on clinical treatment (26). Von Olnhausen et al. focused on evaluating bone flap length, height, and area, but they did not find any significant association between these measurements and clinical outcome (27). In another study, Walz et al. (28) conducted a long-term follow-up of patients with skull defects larger than 14 cm in diameter. They discovered that only 8.3% of patients experienced severe disability, while most had mild to moderate disability. The study conducted by Chung et al. revealed that a larger skull defect diameter resulted in an increased positive rate of patients (mRS < 4) within 3 months after a stroke, when compared to a skull defect diameter greater than 12 cm (29). Additionally, recent findings indicate that a larger area of skull defect plays a crucial role in enhancing neurologic outcomes (30). Our study demonstrates that the size of the skull defect area has a significant impact on the functional outcome of patients who undergo early CP (*p* < 0.1). We found that the skull defect area in patients with a good outcome (mRs ≤ 3) is larger compared to those with a poor outcome (mRs > 3) (138.9 ± 17.3 cm^2^ vs. 126.1 ± 11.1 cm^2^, *p* < 0.001). Previous studies have demonstrated that decompression cannot be effectively achieved by removing small bone flaps. This can result in brain contents becoming trapped at the edge of the bone window, causing incisional herniation and ultimately leading to ischemic necrosis of brain tissue. However, The application of DC can reduce the incidence of encephalocele, and early CP after DC can significantly improve the outcome of patients with encephalocele (26).

Early CP is better than a late CP. Early CP can significantly decrease the long-term complication rate for patients and lead to improved treatment outcomes and prognosis (31). Nasi et al. (32) proposed that performing CP at an earlier stage can expedite the restoration of potential abnormalities caused by the skull defect. These abnormalities may include disturbances in cerebrospinal fluid dynamics, changes in cerebral perfusion, and alterations in oxygen and glucose metabolic rates. Several studies have observed the influence of early CP on neurological function, indicating that it may enhance a patient’s level of consciousness and potentially expedite their recovery (33, 34). A systematic review conducted in 2018 investigated the motor and cognitive changes following CP. The findings revealed that surgeries performed within 90 days resulted in improved motor function. However, there were no significant alterations observed in the Mini-Mental State Examination (MMSE) or memory function (35). A recent systematic review conducted a comparison between early and late CP. The review found no significant difference in baseline neurological function prior to CP. However, it did reveal that patients who underwent early CP (within 3 months after DC) experienced significantly improved clinical outcomes (36). Other studies have also demonstrated that early CP can expedite the process of functional, physical, and cognitive recovery in cases of skull defects (37). In the training group of our study, we observed 90 patients with skull defects who underwent DC. During the 3-month after DC, we assessed the patients’ physical activities and ability to perform daily tasks using the mRs. The study findings revealed that 53 patients (58.9% of the total) were able to independently carry out their daily activities (mRs ≤ 3). These results are consistent with previous studies, highlighting the positive impact of early CP on neurological function.

However, our study had several limitations. It was a small-sample, single-centre study that has not been verified in other hospitals or agencies. Our study included patients with traumatic brain injury, hemorrhagic and ischemic stroke. These patients may have different prognosis and potential for recovery. Since split subgroup analysis was not performed, the conclusions of the study may vary depending on the presence of individuals with different diseases.

Our research found that sex, BMI, past medical history (hypertension, diabetes, and hyperlipidemia), etiology of DC (cerebral hemorrhage, cerebral infarction, and cerebral trauma), side of DC, materials, and operative time was not factor affecting the outcome of early CP. Although these factors may not directly impact the outcome of early CP, they still hold value in terms of providing reference for the development of the final prediction model.

## 5 Conclusion

This study utilized retrospectively collected patient medical record information, combined with LASSO and logistic regression analysis to develop a predictive model for determining the factors that affect short-term outcome of early CP. The model demonstrates a predictive ability sufficient to consider the further development of the model and its clinical application.

## Data availability statement

The raw data supporting the conclusions of this article will be made available by the authors, without undue reservation.

## Ethics statement

The studies involving humans were approved by the Ethics Committee of Xi’an Gaoxin Hospital. The studies were conducted in accordance with the local legislation and institutional requirements. Written informed consent for participation was not required from the participants or the participants’ legal guardians/next of kin because this is a retrospective study.

## Author contributions

ZY: Writing – review & editing, Writing – original draft, Visualization, Validation, Supervision, Software, Resources, Project administration, Methodology, Investigation, Formal analysis, Data curation, Conceptualization. XL: Writing – review & editing, Writing – original draft, Visualization, Validation, Supervision, Software, Project administration, Methodology, Investigation, Formal analysis, Data curation. BX: Writing – review & editing, Software, Data curation. CX: Writing – review & editing, Methodology. YW: Writing – review & editing, Methodology. HC: Writing – review & editing, Software, Methodology. DS: Writing – review & editing, Formal analysis. SG: Writing – review & editing, Writing – original draft, Visualization, Validation, Supervision, Software, Resources, Project administration, Methodology, Investigation, Formal analysis, Data curation, Conceptualization.
